# Effects of lactoferrin on intestinal flora of metabolic disorder mice

**DOI:** 10.1186/s12866-022-02588-w

**Published:** 2022-07-22

**Authors:** Li Li, Chunli Ma, Hurilebagen, Hong Yuan, Ruiping Hu, Wuji Wang, Weilisi

**Affiliations:** 1 Basic Medical College of Inner Mongolia Medical University, Hohhot, 010110 China; 2 Mongolian Medicine College of Inner Mongolia Medical University, Hohhot, 010110 China

**Keywords:** Lactoferrin, Metabolic disorders, Intestinal flora

## Abstract

To study the mechanism of lactoferrin (LF) regulating metabolic disorders in nutritionally obese mice through intestinal microflora. Twenty-one male C57BL/6 mice were randomly divided into 3 groups: control group, model group and LF treatment group. The mice in control group were fed with maintenance diet and drank freely. The mice in model group were fed with high fat diet and drank freely. The mice in LF treatment group were fed with high fat diet and drinking water containing 2% LF freely. Body weight was recorded every week. Visceral fat ratio was measured at week 12. Blood glucose and serum lipid level were detected by automatic biochemical analyzer. The gut microbiota of mice was examined using 16 s rRNA sequencing method. LF treatment significantly reduced the levels of visceral adipose ratio, blood glucose, triglyceride, total cholesterol and low-density lipoprotein cholesterol (LDL-C) in high-fat diet mice (*p* < 0.05). It can be seen that drinking water with 2% LF had a significant impact on metabolic disorders. At the same time, the *Firmicutes/Bacteroidetes* ratio(F/B) of LF treated mice was decreased. The abundance of *Deferribacteres, Oscillibacter, Butyricicoccus, Acinetobacter* and *Mucispirillum* in LF treatment group were significantly decreased, and the abundance of *Dubosiella* was significantly increased (*p* < 0.05). In the LF-treated group, the expression levels of glucose metabolism genes in gut microbiota were increased, and the expression levels of pyruvate metabolism genes were decreased. It can be seen that metabolic disorders were related to intestinal flora. In conclusion, LF regulates metabolic disorders by regulating intestinal flora.

**Supplementary Information:**

The online version contains supplementary material available at 10.1186/s12866-022-02588-w.

Obesity is a metabolic disease in which excess fat accumulates in the body and leads to metabolism disorders [1]. Studies have confirmed that adipose cells are the internal energy reserve organs of the body and have functions similar to endocrine organs [2]. When there is more adipose tissue in visceral organs, it plays an important role in the occurrence and development of many metabolic diseases [3]. Metabolic syndrome (Met S) is highly related to the excessive accumulation of visceral adipose tissue (VAT) [4]. Metabolic disorders not only related to genetic and environmental factors, but also to the intestinal microbes of the host [5].

The number of genes encoded by intestinal flora is more than 100 times that of the human body itself [6]. Once the gut flora is out of whack, inflammation, obesity and cancer can follow. In 2004, a research group of Professor Jeffery I. Gordon, confirmed for the first time that intestinal flora can regulate the accumulation of fat in the host as an environmental factor [7]. The intestinal flora of human body is mainly composed of bacteria of 9 phyla, among which Bacteroidetes and Firmicutes occupy an absolute dominance, and their proportion is closely related to the occurrence of obesity [8]. It was found that the abundance of Bacteroidetes in the intestinal tract of obese mice decreased by 50%, while the proportion of Firmicutes increased [9]. Studies have confirmed that [10] the germ-free mice receiving the intestinal flora of obese mice were significantly heavier than those receiving the intestinal flora of emaciated mice after 2 weeks respectively. Emerging evidence suggests that the gut microbiome, commensal microbes that colonize the gastrointestinal tract, also play a significant role in type 2 diabetes mellitus (T2DM) pathogenesis [11]. More and more research evidence indicate that intestinal flora plays an extremely important role in the occurrence and development of metabolic disorders.

Lactoferrin (LF) is an iron-binding glycoprotein. LF not only participates in iron transport, but also has many biological functions, including antibacterial, anti-inflammatory, anti-tumor and immunomodulatory functions [12]. LF has also been reported to balance energy metabolism, reduce body mass and improve obesity [13]. Xiong et al. found that LF intervention for 15 weeks could significantly reduce high-fat diet (HFD)-induced obesity and body fat in mice, and LF attenuates HFD-induced hepatic steatosis and lipid metabolic dysfunctions by suppressing hepatic lipogenesis and down-regulating inflammation in C57BL/6 J mice [14]. The purpose of this study was to explore the potential of preventing and treating metabolic diseases by supplementing with LF, and to try to explain the mechanism from the perspective of gut microbiota.

## Materials and methods

### Animals and diets

3-week-old male C57BL/6 mice were purchased from SPF Biotechnology Co., LTD. (License number: SCXK (Beijing) 2019–0010) (Beijing, China). The mice lived in an environment with constant temperature of 23 ± 2 °C and 60% humidity. After 1 week of acclimatization, 21 animals were randomly divided into 3 groups: (1) control group (K group, *n* = 7), (2) HFD group (M group, *n* = 7), (3) HFD plus LF (Y2 group, *n* = 7). Control diet (10% calories from fat, D12450J) and HFD diet (60% calories from fat, D12492) were purchased from Xietong biology Co., LTD. (Jiangsu, China). LF was obtained Tatua Co-operative Dairy Company, Limited (Tatua, New Zealand). LF has a purity of 95% and iron saturation of 15%. According to the literature, LF was dissolved 2 g/100 mL in distilled water [15]. The drinking water was changed every day. The animals had free access to water and diets. The experiment lasted for 12 weeks. The body weight of mice was recorded weekly. All procedures were carried out in accordance with the Guidelines for Animal Care and Use approved by the Medical Ethics Committee of Inner Mongolia Medical University.

### Collection of samples

One day before the end of the experiment, the second fresh feces of the mice were collected on the aseptic operating table after the tail of the mice were pulled to stress the defecation, and quickly transferred to the aseptic eppendorf tube for freezing storage at − 80 °C. After fecal samples were collected, the animals were fasted for 12 h and blood was collected under anesthesia with sodium pentobarbital solution. Serum was separated by centrifugation and stored at − 80 °C. Adipose tissue around visceral organs was collected and weighed. Recorded date was used to calculate visceral adipose ratio (visceral adipose ratio = visceral adipose tissue weight (g)/mouse body weight (g)).

### Serum biochemical determination

The serum glucose, triglyceride (TG), total cholesterol (TC), high-density lipoprotein (HDL)-cholesterol (HDL-C) and low-density lipoprotein (LDL)-cholesterol (LDL-C) were analyzed using commercial enzymatic assay kits (AIDISHENG, Jiangsu, China). Insulin levels were measured using an enzyme-linked immunosorbent assay kit (Enzyme-linked Biotechnology, Shanghai, China).

### Intestinal flora detection

Genomic DNA was extracted from feces by sodium dodecyl sulfate, and the purity and concentration of DNA were detected by agarose 1% gel electrophoresis. An appropriate amount of genomic DNA was taken and diluted to 1 ng/μl in sterile water in a centrifuge tube.

The template diluted DNA sample(1 ng/μl) and Phusion® High-Fidelity PCR Master Mix with GC Buffer (New England Biolabs) was used to amplify the 16S rRNA V4 gene marker. Each DNA sample of the bacterial 16S rRNA gene was amplified with primers 515F (5′-GTTTCGGTGCCAGCMGCCGCGGTAA-3′) and 806R (5′-CAGATCGGACTACHVGGGTWTCTAAT-3′). The amplicons obtained by PCR were analyzed on 2% agarose gel electrophoresis, and a band of a desired size was purified using a QIAquick gel extraction kit (QIAGEN, Germany). TruSeq® DNA PCR-free Sample Preparation Kit was used for library construction. The qualified library was submitted to the second-generation NovaSeq6000 platform sequencing.

### Bioinformatics analysis

The raw pyrosequencing reads obtained from the sequencer were denoised using FLASH software (V1.2.7). According to the barcode and primer sequences, the resulting pyrosequencing reads were filtered referring to QIIME(V1.9.1) [16]. Tags quality control process. Finally, the chimera sequences were detected and excluded from the denoised sequences. The final Effective Tags were obtained. The effective tags reads were clustered into operational taxonomic units (OTUs). In order to study the species composition of each sample, the samples were clustered. OTUs that reached a 97% nucleotide similarity level were used for clustering analysis and species annotation. QIIME software (Version 1.9.1) was used for alpha diversity (Shannon, Simpson), richness (ACE and Chao1), Good’s coverage, Observed-OTUS, PD-whole-tree. R software (Version 2.15.3) was used rarefaction curve analyses. Differences in alpha diversity index between groups were analyzed by parametric and nonparametric tests, respectively. The t-test and Wilcox test methods were used when only two groups were present. Tukey’s test and Agricolae’s Wilcox test were used when there were more than two groups.

Beta diversity analysis mainly used the binary Jaccard algorithm to calculate the distance among samples to obtain the beta value between samples. Based on the distance matrix obtained by beta diversity analysis, the differences of species diversity among samples were further demonstrated. Principal component analysis (PCA) was performed using R language tools (ade4 package and ggplot2 software). Principal co-ordinates analysis (PCoA) was performed using R language tools (WGCNA package, stats package and ggplot2 software). Then, linear discriminant analysis (LDA) effect size (LEfSe) tools (LDA score filter value to 4) were used the Wilcox test function of the R language STATS package to estimate the impact of the abundance of each component (species) on the effect of the difference between components [17].

PICRUSt (Version 1.1.4) software was used to compare the species composition information obtained from 16S sequencing data, infer the composition of functional genes in the sample, and determine the functional differences between different groups [18]. Kyoto Encyclopedia of Genes and Genomes (KEGG) ortholog database (tertiary metabolic pathway) were used to analyze for Tax4 Fun function prediction [19], and the changes in metabolic pathways of functional genes of microbial communities between different groups [20, 21].

### Statistics analysis

Statistical analysis was performed using SPSS22.00 software. Independent T tests and Wilcox T tests were used for continuous variables. All significance tests were two-sided tests, and *p* < 0.05 or adjusted *p* < 0.05 was considered statistically significance.

## Results

### Effects of LF on body weight，visceral adipose ratio and feed efficiency

**Fig. 1:**
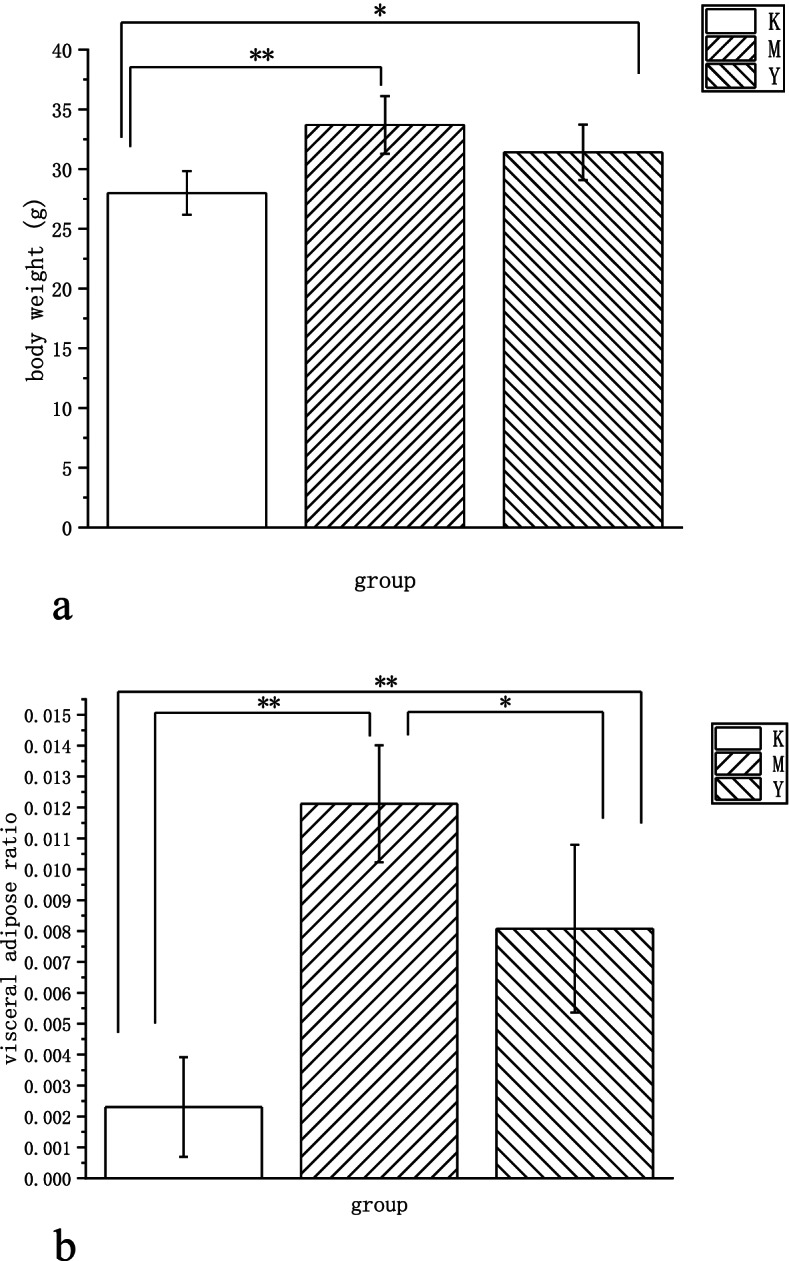
**a** Weight differences in mice at week 12. Data are presented as mean ± SD (*n* = 7) (SPSS 11.0). Abbreviations: K, the control group; M, the model group; Y2, the LF-treated group. * *p* < 0.05, ** *p* < 0.01. **b** Visceral adipose tissue differences in mice at week 12. Data are presented as mean ± SD (*n* = 7) (SPSS 11.0). Abbreviations: K, the control group; M, the model group; Y2, the LF-treated group. * *p* < 0.05, ** *p* < 0.01

Body mass for all mice was nearly identical at the beginning of experiment. As indicated in Fig. 1a, After12 weeks, Y2 and M groups gained weight rapidly. The 12-week HFD-induced body mass of Y2 and M groups was significantly higher than the standard diet of K group (average weight, K = 28 g, M = 33.7 g, Y2 = 31.4 g). The weight of M group was 20% higher than that of K group (Obesity model standard: the weight of mice in the model group exceeds 20% of the weight of mice in the normal group). There was no significant difference in body weight between Y2 group and M group. The body weight increasing of M and Y2 group was accompanied by a notable white fat accumulation, including epididymal, perinephric, and mesenteric adipose.

It was found that visceral adipose ratio of mice in group K was significantly different from that in groups M and Y2(*P* = 0.00). Visceral adipose ratio of mice in Y2 group decreased and was significantly different from that in M group (*P* = 0.032). Together these data indicated that the model of obesity in mice was successfully built (Fig.1b).

**Fig. 2:**
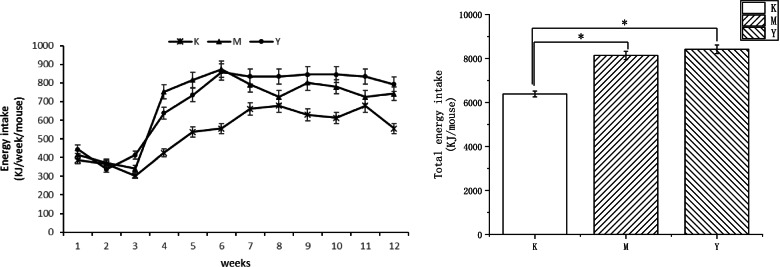
Effects of LF on energy intake in mice

LF reduced feed efficiency in high-fat-fed mice. The energy intake of the Y group was higher than that of the M group, but the difference was not significant (Fig. 2). The ratio of visceral adipose in the Y group was significantly lower than that of the M group (Fig. 1b).

### Effect of LF on serum biochemical indices in high-fat-fed induced obese mice

A long-term, high-fat-fed is often associated with disordered lipid and glucose metabolism. Compared with K group, blood glucose, TG, LDL-C were significantly increased in M group (*P* < 0.05). Compared with M group, blood glucose, TG, TC and LDL-C in Y2 group were significantly decreased (*P* < 0.05) (Table 1).

**Table 1:** Effect of LF on serum biochemical indices in mice

Serum biochemical indices	K group	M group	Y2 group
blood glucose (mmol/L)	5.28 ± 0.06^ac^	6.80 ± 0.05^ab^	5.92 ± 0.06^bc^
insulin (mmol/L)	19.20 ± 1.00	20.38 ± 1.09	19.59 ± 0.73
TG (mmol/L)	0.20 ± 0.06^ac^	0.38 ± 0.02^ab^	0.28 ± 0.01^bc^
TC (mmol/L)	4.57 ± 0.02^c^	4.57 ± 0.05^b^	3.71 ± 0.03^bc^
LDL-C (mmol/L)	2.17 ± 0.02^ac^	3.06 ± 0.02^ab^	1.84 ± 0.02^bc^
HDL-C (mmol/L)	2.40 ± 0.04^ac^	1.51 ± 0.00^ab^	1.88 ± 0.04^bc^

Note: All data were expressed as mean ± standard deviation. ^a^ represents significant difference between group K and group M, ^b^ represents significant difference between group M and group Y2, and ^C^ represents significant difference between group K and group Y2

### Effect of LF on intestinal microflora in high-fat diet mice

Sequencing data total 1,691,261 original sequences were obtained from 21 samples, and the sequence length was about 418 bp. After double-end reads splicing and filtering, a total of 1,539,258 clean tags were generated. Taxonomic annotation of OTUs was obtained with SSU rRNA database [22] of SILVA138 [23]. A total of 14,835 OTUs were obtained. Fast multiple sequence alignment was performed using MUSCLE [24] (Version 3.8.31) software. Coverage approached 99.0% for all sequences in the three groups, indicating good sequencing depth for investigation of the depression associated fecal microbiota.

**Fig. 3:**
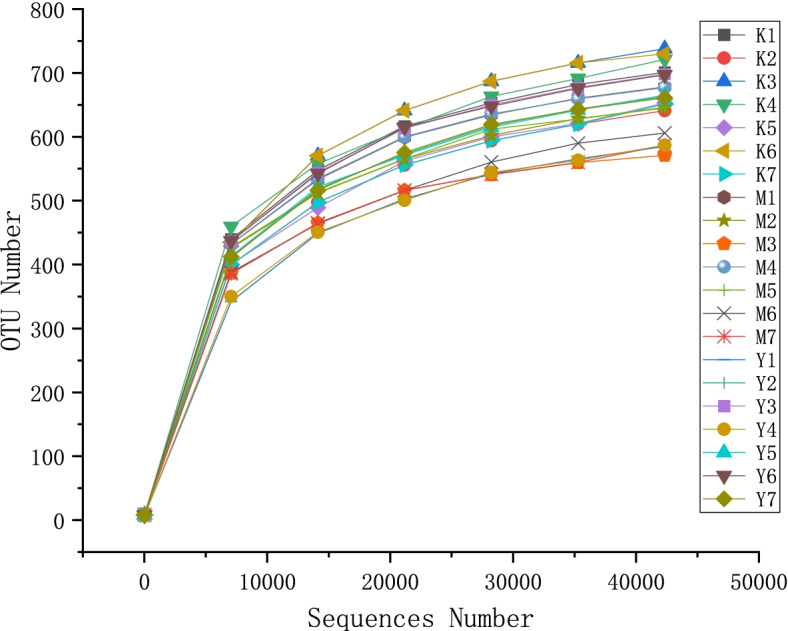
Rarefaction curves of fecal sample. The number of OTU tended to be stable after the sequence was over 40,000. Abbreviations: K1-K7, control group; M1-M7, model group; Y1-Y7, LF-treated group

The dilution curve (Fig. 3) showed that the detected species increased with the increase of sample sequencing amount, and the number of OTU tended to be stable after the sequence was over 40,000.

### Alpha diversity comparisons among three groups

**Fig. 4:**
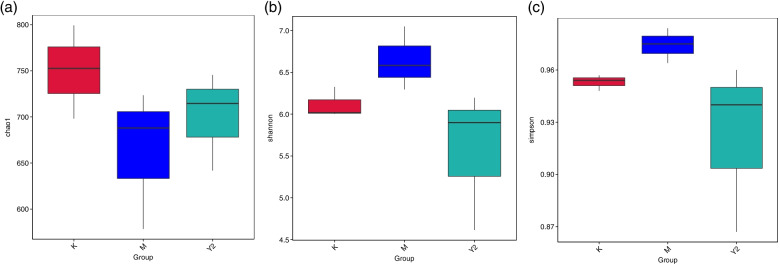
Comparison of the flora alpha diversity indices. **a** Indices of Chao 1, **b** Indices of Shannon **c** Indices of Simpson. There were not significant differences among the groups. The results showed that the diversity of intestinal microflora of mice in group M was higher than that in group K, and the diversity of intestinal microflora of mice in group Y2 was closer to that in group K. Abbreviations: K, the control group; M, the model group; Y2, the LF-treated group

Alpha diversity was used to reflect the richness and diversity of species in samples. The indexes of Chao 1, Shannon and Simpson were shown in Fig. 4. The differences were not significant among the groups. However, the box diagram intuitively showed that the diversity of intestinal microflora of mice in group M was higher than that in group K, and the diversity of intestinal microflora of mice in group Y2 was closer to that in the healthy group.

### Beta diversity comparisons among three groups

**Fig. 5:**
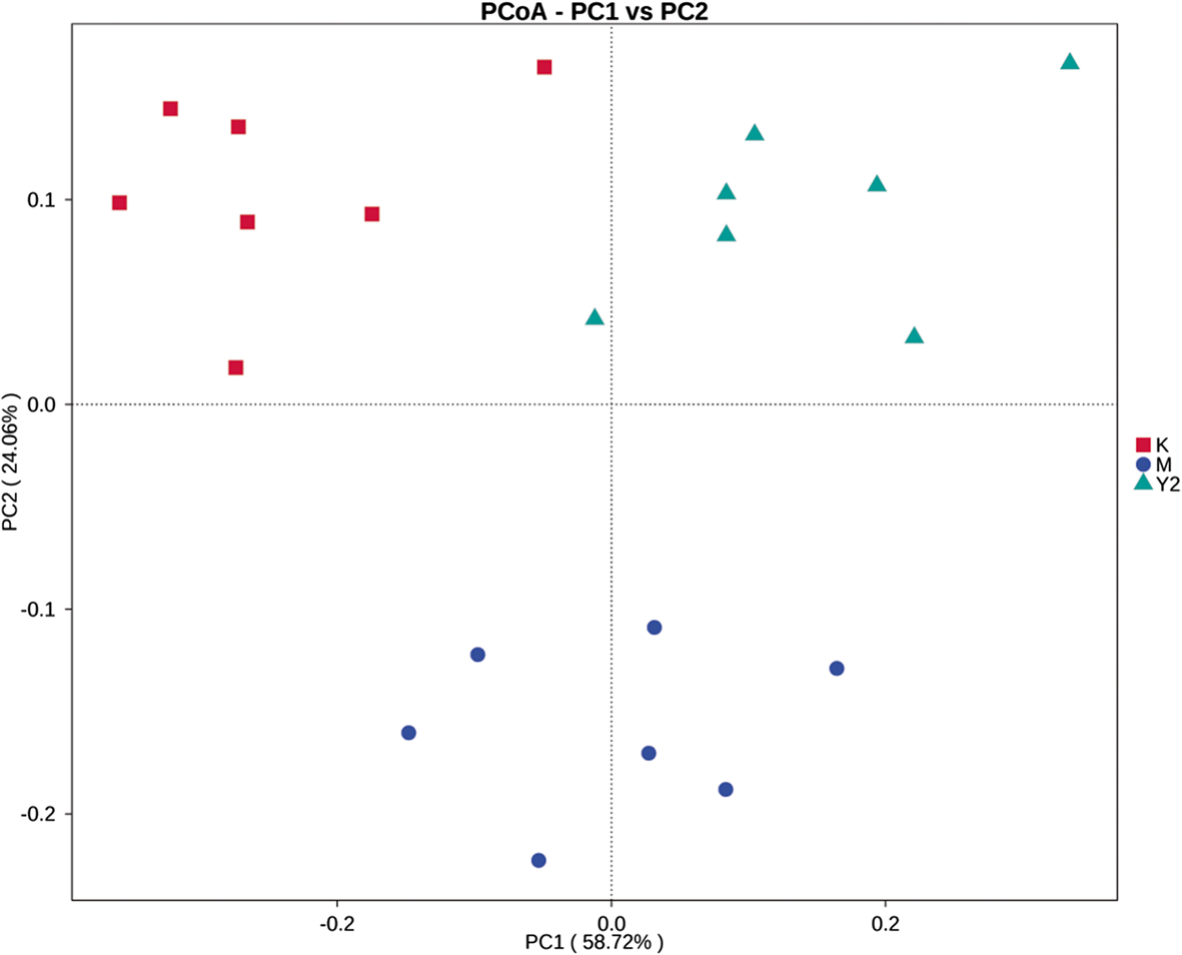
Beta diversity of P CoA. There were significant differences in the composition and structure of intestinal flora among the three groups. Group K was clustered in the upper left part of the main horizontal axis. The Y2 group was clustered in the upper right part of the main horizontal axis. Group M gathered in the lower part of the main horizontal axis. Abbreviations: square, control group; round, model group; triangle, LF-treated group. Note: K control group， M model group，Y experimental group

Beta diversity was used to compare the similarity of species diversity among different groups. The binary Jaccard algorithm was used to calculate beta diversity. P CoA analysis showed that the composition and structure of intestinal flora in the three groups were significantly different (Fig. 5). Mice in the control group formed distinct clusters of microbial species on the upper left of the main horizontal axis. Mice in group Y2 formed a distinct cluster of microbial species on the upper right of the main horizontal axis. Mice in group M formed obvious microbial species cluster in the lower part of the main horizontal axis.

### Intestinal microflora structure among three groups

**Fig. 6:**
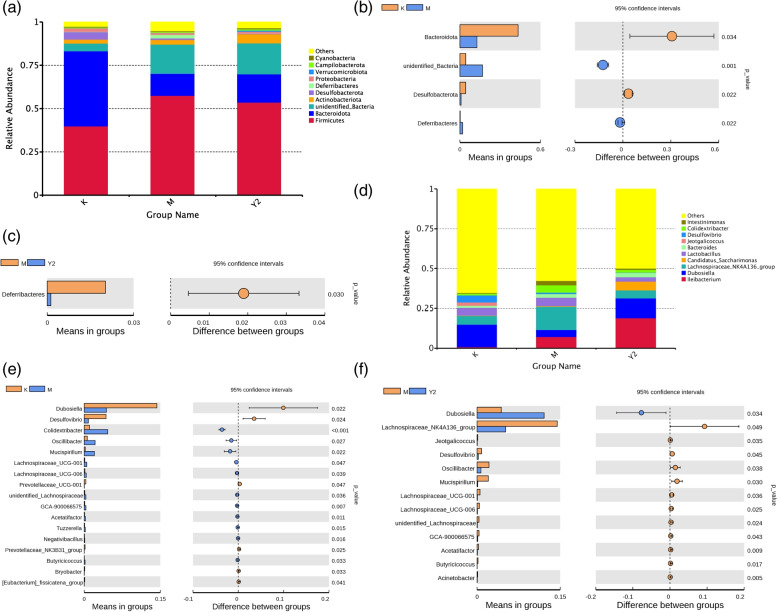
Effects of LF on the abundance of ileal microbiota in mice, *n* = 7. **a** Phylum-level composition. The ratio of Firmicutes and Bacteroidetes (F/B) decreased in Y2 group relative to M group. **b** t-test analysis at the phylum level, the abundance of *Deferribacteres* in group M was significantly higher than that in group K(*P* = 0.022). **c** t-test analysis at the phylum level, the abundance of *Deferribacteres* in group M was significantly higher than that in group Y2(*P* = 0.030). **d** Genus-level composition. **e** t-test analysis at the genus level, the abundance of *Dunaliella* in group M was significantly lower than that in group K (*P* = 0.022). **f** t-test analysis at the genus level, the abundance of *Dunaliella* in group Y2 was significantly higher than that in group M(*P* = 0.034). Abbreviations: K, the control group; M, the model group; Y2, the LF-treated group

There is growing evidence that the gut microbiome plays an important role in the development of obesity and obesity-related complications. The results of relative species abundance at phylum level analysis showed that relatively high proportions were: *Fimicutes, Bacteroidota (Bacteroidetes), unidentified-Bacteria, Actinobacteriota (Actinobacteria), Desufobacterota* and *Deferribacteres.* The abundances varied greatly at phylum level among different groups (Fig. 6). The abundance of *Firmicutes* decreased in Y2 group relative to M group, and the abundance increased in M group relative to K group. The abundance of *Bacteroidetes* increased in Y2 group relative to M group and decreased in M group relative to K group. The ratio of *Firmicutes* and *Bacteroidetes* (F/B) increased in M group relative to K group, and decreased in Y2 group relative to M group. After independence test, normality test, and homogeneity of variance test, t test can be performed on two independent samples. Results in groups with mean abundance less than 0.001 (default 0.001) are first filtered. Species that differed significantly between groups were identified at each taxonomic level (*p*-value < 0.05). t-test analysis showed that there were significant differences in the abundance of *Deferribacteres* among the different groups. It was significantly lower in Y2 group than in M group, and significantly higher in M group than in K group (*p* < 0.05).

The results of relative species abundance at genus level analysis showed that relatively high proportions were: *Ileibacterium, Dubosiella, Lachnospiraceae_NK4A136_group, Candidatus, Saccharimonas, Lactobacillus, Bacteroides.* T-test analysis showed that the abundance of *Dubosiella, Oscillibacter, Butyricicoccus, Acinetobacter* and *Mucispirillum* were significantly different in M and Y2 groups. The abundance of *Dubosiella* was significantly higher in Y2 group than in M group, and it was significantly lower in M group than in K group (p < 0.05). The abundance of *Oscillibacter, Butyricicoccus, Acinetobacter* and *Mucispirillum* were significantly lower in Y2 group than in M group (p < 0.05).

### The correlation of gut microbiota among the three groups

**Fig. 7:**
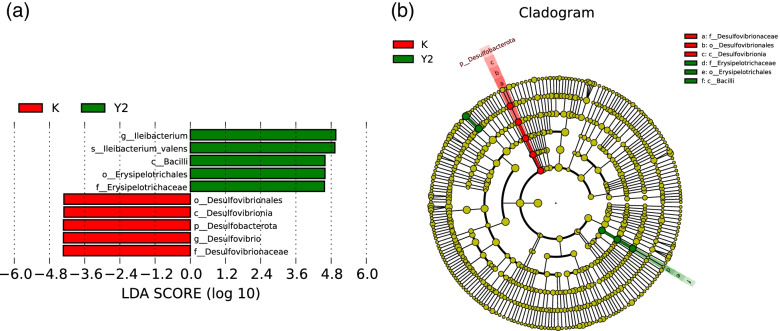
High-dimensional biomarkers (LDA value distribution histogram revealed by LEfSe software). **a** Histogram of LDA analysis. When species with LDA Score > 4 are statistically different, the length of the histogram (LDA Score) represent the impact size of the different species. **b** The distribution difference of flora was analyzed by LEfSe. Evolutionary branching trees from the inside out in a clade represent the level of phylum, class, order, family, genus (or species). Classification of species in which red/green nodes play an important role in healthy controls. The yellow nodes indicate species that are not significantly different. Abbreviations: K, the control group; Y2, the LF-treated group

LEfSe was used to interpret high-dimensional biomarkers of fecal samples, and find statistically different Biomarkers among different M and Y2 groups (K, M, Y2). Statistically different Biomarkers were displayed in the LDA value distribution histogram (Fig.7a). There were no significantly different species in group M, so this group was deleted. In Y2 group, the abundance was significantly reduced, including *Desulphurvibrio, Desulfovibrionia, Desulfovibrionales, Desulfovibrionaceae, Desulfovibrio.* There was a significant increase in the abundance, including *Erysipelotrichales, Erysipelotrichaceae, Bacilli, Ileibacterium, Ilebacterium_valens.* It was considered significant in Kruskal–Wallis and pairwise Wilcoxon tests when *p* < 0.05.

Evolutionary branching trees from the inside out in a clade represent the level of classification from phylum to genus (or species). The red nodes represent the microbial groups that play an important role in group K. The green nodes represent the microbial groups that play an important role in group Y2. The yellow nodes indicate species that are not significantly different (Fig. 7b). The evolution of marker species has the following relationships: *Firmicutes-Bacilli-Erysipelotrichia-Erysipelotrichales-Erysipelotrichaceae-Ileibacterium-Ilebacterium_valens; Desulfobacterota-Desulfovibrionia-Desulfovibrionales-Desulfovibrionaceae-Desulfovibrio.*

### Functional properties predicted by PICRUSt of LF on intestinal microflora function in high-fat diet mice

**Fig. 8:**
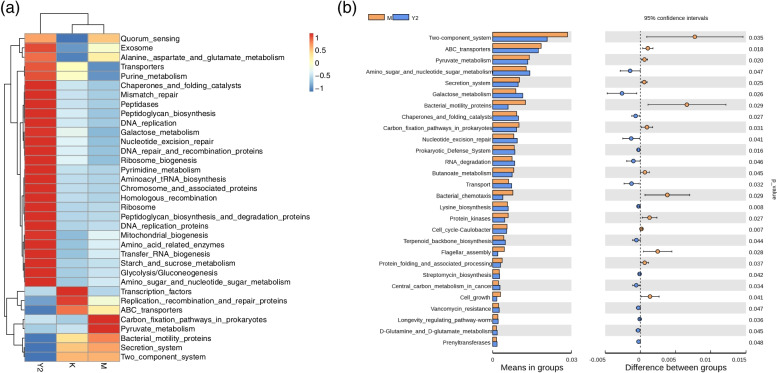
The KEGG prediction of intestinal flora. **a** Annotations the functional gene. **b** Expression levels analysis by t-test. Significant difference when *p* < 0.05. Abbreviations: K, the control group; M, the model group; Y2, the LF-treated group

The PICRUSt software was used to compare the species composition information obtained from 16S sequencing data to infer the functional gene composition in three groups. Annotations classified by tertiary KEGG pathway (Fig.8a). Many functional pathways were showed significant differences between groups by t-test analysis (Fig. 8b). Compared with group M, the expression levels of pyruvate metabolism and carbon fixation pathway genes in prokaryotes of Y2 group were significantly decreased, and the gene expression levels of galactose metabolism, amino sugar and nucleotide sugar metabolism genes were significantly increased (*p*<0.05) (Fig.8).

## Discussion

In this study, C57 mice were fed a HFD to induce obesity (a metabolic disease), while intervening with LF. The results showed that LF intervention decreased visceral adipose tissue, serum blood glucose and serum lipid content of these mice, and LF reduced feed efficiency in high-fat-fed mice. It can be seen that LF has regulated the metabolism of mice in this experiment. This result is similar to previous articles [25, 26]. However, the experimental time, LF supplementation method and dose are different, so that the experimental results are slightly different from those of the reference. In the previous article, LF was administered at 100 mg per body weight for 15 weeks. In this experiment, LF was provided as a 2% aqueous solution for 12 weeks ad libitum. A variety of experimental conditions contributed to the discrepancies in the experimental results. The present study also found that LF intervention improved the imbalance of gut microbiota in HFD mice, reduced the diversity of gut microbiota, and made the microbiota structure closer to healthy mice. The chao1 index showed that HFD decreased the total number of gut flora in mice compared with normal diet mice, and LF intervention alleviated the decline in the number of intestinal flora species in mice, but the difference was not significant. The Simpson and Shannon index showed that high-fat diet increased the diversity and uniformity of species distribution in the intestinal microflora of mice, and LF intervention improved the changes of intestinal microbiota in mice caused by high-fat diet. P CoA analysis showed that the intestinal flora of the control group, model group (HFD) and LF intervention group (HFD + LF) formed obvious clusters of microbial species at different positions on the abscissa axis. It can be seen that the composition and structure of gut microbes were different.

At phylum-level, the abundance varied widely among the three groups. The model group had an increase in *Firmicutes* and a decrease in *Bacteroidetes* relative to the control group, making their ratios increase. The abundance ratio of *Firmicutes/Bacteroidetes* decreased in the LF intervention group. The ratio of *Firmicutes* to *Bacteroidetes* is generally regarded as a biological marker for the existence of obesity [27]. The reduction of this ratio can regulate abnormal short-chain fatty acid metabolism, inhibit chronic mild inflammatory response [28], promote the secretion of glucagon like peptide (GLP-1) [29], improve Insulin resistance. There were significant differences in the abundance of *Deferribacteres* among the different groups. It was significantly lower in Y2 group than in M group, and significantly higher in M group than in K group. The abundance of the *Deferribacteres* is a common phenomenon in obesity, which is positively linked with the pro-inflammatory factors interleukins-6, tumour necrosis factor-alpha, and interleukins-17A, causing aggravation of inflammation in obesity [30]. LF may protect the integrity of the gut barrier by downregulating F/B ratio, inhibiting the overgrowth of inflammation-related bacteria, and repairing the adverse changes in gut microbiota induced by a high-fat diet.

At the genus level by LEfSe analysis, the abundance of *Dunaliella* in the LF-treated group was significantly increased (*p* < 0.05). *Dubosiella* is used as a patented probiotic for modulating weight loss and preventing metabolism and immunity associated diseases, such as obesity, diabetes, metabolic syndrome, and abnormal lipid metabolism [31]. There were significantly decreased abundances of *Oscillibacter, Butyricicoccus, Acinetobacter* and *Mucispirillum* in the LF treated group. (*p* < 0.05). These commensal bacteria are able to induce intestinal inflammation or pathology in diet-induced obesity, diabetes, or any change in the gut environment [32].

We found that *Ileibacterium -valens* was significantly increased in the gut microbiota of mice in the LF intervention group compared to the control group. The study [33] had confirmed that abundance of *Erysipelotrichaceae- Ileibacterium* was associated with obesity. It may affect the host metabolism and inflammatory diseases and is closely related to obesity [34]. *Ileibacterium valens* is a recently discovered anaerobic bacteria [35]. More research on *I. valens* awaits further study.

KEGG pathway analysis showed that there were significant differences in the expression levels of each functional gene among the three groups. The expression levels of genes for glucose metabolism (galactose metabolism, starch and sucrose metabolism, glycolysis and gluconeogenesis, amino sugar and nucleotide sugar metabolism) were increased in the LF intervention group. LF treatment reduced the expression levels of pyruvate metabolism and prokaryotic carbon fixation pathway genes. In conclusion, LF treatment increased the expression levels of glucose metabolism genes in mice, while the expression levels of pyruvate metabolism genes decreased, so the amount of pyruvate in the gut increased, which led to an increase in pyruvate entering the blood. Pyruvate can enhance glucose metabolism and fat metabolism, thus reducing energy accumulation and inhibiting adipogenesis [36]. This is consistent with the results that LF reduces blood sugar and blood lipids. However, the pathway of action requires further study.

There are still shortcomings in this study. Due to the microbiota intervention study maintained for 12 weeks, the mice were in a growing state throughout the experiment from 3 to 15 weeks of age. The effects of LF in mice may not only suppress obesity but also promote growth. Therefore, there was little difference in body weight between the model group and the LF intervention group. We plan to further investigate the mechanism by which LF regulates metabolism.

## Conclusions

In this study, we used LF to intervene in the high-fat-induced metabolic disorder model of mice, and compared the metabolic indexes and intestinal microbial community indexes of mice in the obesity model group and the LF treated group. The results showed that the feed efficiency, blood glucose, blood lipid and visceral adipose rate in LF treatment group were decreased, and the intestinal microflora and function of mice were changed. Therefore, we infer that LF regulates metabolism by regulating the gut microbiome. LF may open up new possibilities for the prevention and treatment of metabolic disorders.

## Supplementary Information

**Additional file 1.**

**Additional file 2.**

**Additional file 3.**

## Data Availability

The data that support the findings of this study are available from the supplementary information files. Supplementary 1: body weight, visceral adipose ratio, rarefaction curves; Supplementary 2: Beta diversity; Supplementary 3: KEGG pathway annotation.
