# Supplementary figures and images for: Effects of lactoferrin on intestinal flora of metabolic disorder mice
Source: BMC Microbiol. 2022 Jul 22;22:181. doi: 10.1186/s12866-022-02588-w (PMC9306164; doi:10.1186/s12866-022-02588-w)

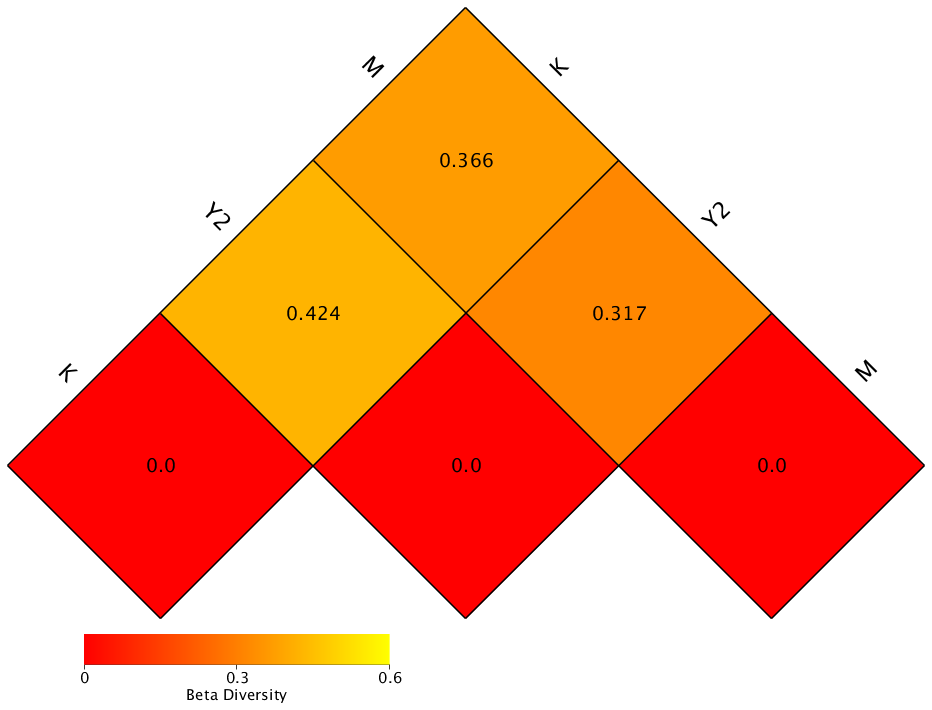

Supplement: Supplementary file 2 — Additional file 2. [file 12866_2022_2588_MOESM2_ESM.png]

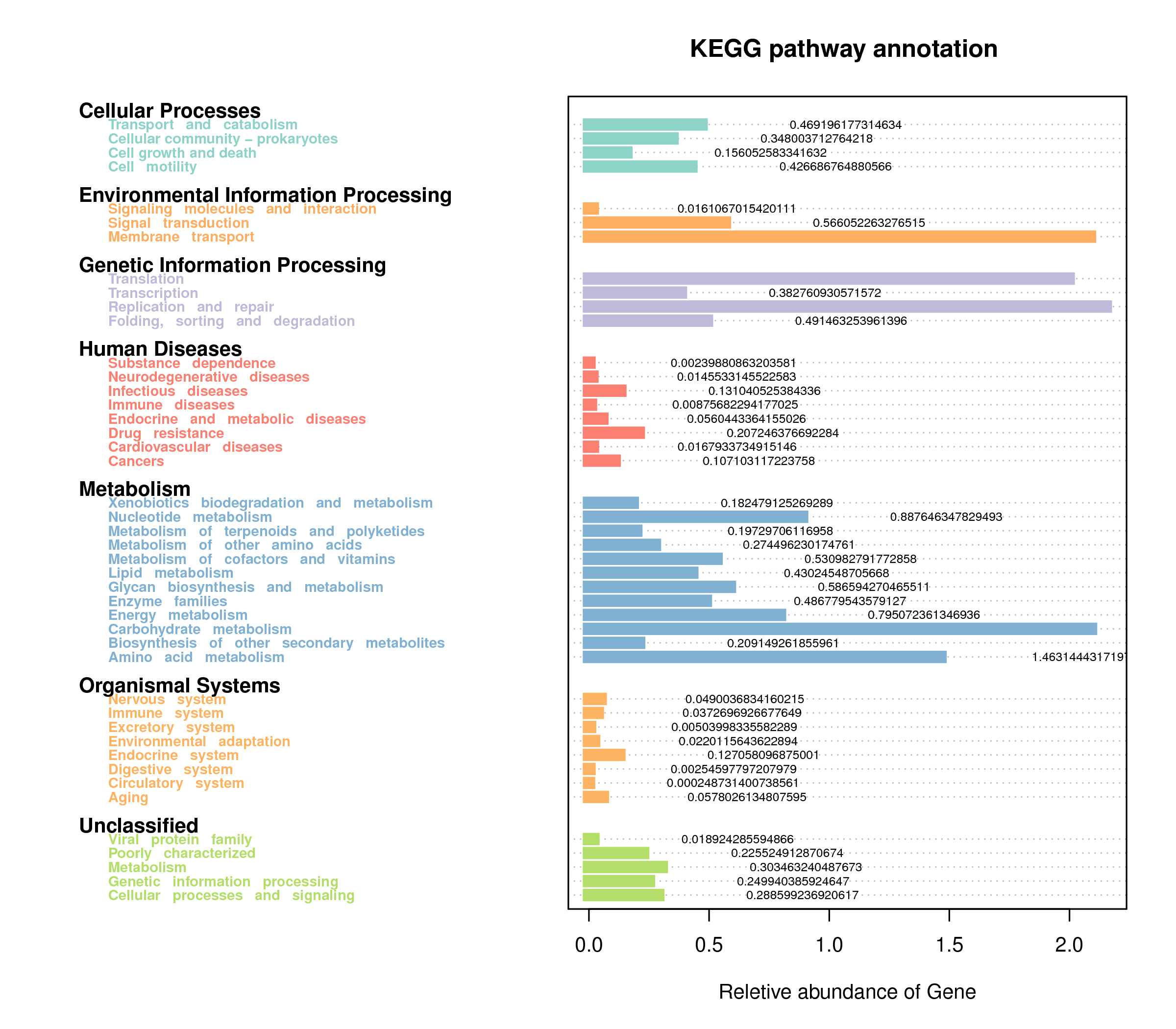

Supplement: Supplementary file 3 — Additional file 3. [file 12866_2022_2588_MOESM3_ESM.png]
